# Methane Ameliorates Lipopolysaccharide-Induced Acute Orchitis by Anti-inflammatory, Antioxidative, and Antiapoptotic Effects via Regulation of the PK2/PKR1 Pathway

**DOI:** 10.1155/2020/7075836

**Published:** 2020-08-18

**Authors:** Chao Huang, Wenbo Zhang, Aijun Sun, Xi Zhang, Jinping Guo, Ruijuan Ji, Liang Qiao, Xuejun Sun, Dongbao Zhao

**Affiliations:** ^1^Department of Anatomy, Institute of Biomedical Engineering, Naval Medical University, #800 Xiangyin Road, Shanghai 200433, China; ^2^Department of Rheumatology and Immunology, Changhai Hospital Affiliated to the Naval Medical University, Shanghai 200003, China; ^3^Department of Navy Aeromedicine, Naval Medical University, #800 Xiangyin Road, Shanghai 200433, China

## Abstract

**Objective:**

The present study is aimed at investigating the anti-inflammatory, antioxidative, and antiapoptotic effects of methane on lipopolysaccharide- (LPS-) induced acute orchitis and its potential mechanisms.

**Methods:**

Adult male rats were intraperitoneally (i.p.) injected with methane-rich saline (MS, 20 mL/kg) following LPS (5 mg/kg, i.p.). The survival rate was assessed every 12 h until 72 h after LPS induction, and surviving rats were sacrificed for further detection. The wet/dry (*W*/*D*) ratio was determined, and testicular damage was histologically assessed. Inflammatory cytokines in the testes and serum, including interleukin-1*β* (IL-1*β*), IL-6, IL-10, and tumor necrosis factor-*α* (TNF-*α*), were measured using ELISA and RT-qPCR. Oxidative stress was evaluated by the level of superoxide dismutase (SOD) and malondialdehyde (MDA). Testicular apoptosis was detected via TUNEL staining. The expression of prokineticin 2 (PK2)/prokineticin receptor 1 (PKR1) was also analyzed using RT-qPCR, western blotting, and immunohistochemistry.

**Results:**

It was found that methane significantly prolonged rat survival, decreased the W/D ratio, alleviated LPS-induced histological changes, and reduced apoptotic cells in the testes. Additionally, methane suppressed and promoted the production of pro- and anti-inflammatory cytokines, respectively. Furthermore, methane significantly increased SOD levels, decreased MDA levels, and decreased testicular expression of PK2 and PKR1. Therefore, methane exerts therapeutic effects on acute orchitis and might be a new and convenient strategy for the treatment of inflammation-related testicular diseases.

## 1. Introduction

It has been reported that infertility, including male infertility, affects ~16% of couples worldwide and is relatively prevalent in clinic [1]. Recent researches have demonstrated that infection and inflammation of the testes are one of the major contributors to male infertility, accounting for ~15% of cases of male infertility [2]. Therefore, the investigation of effective treatment for orchitis plays a vital role in the therapy of male infertility.

After years of exploration, it has been proved that the pathogenesis of orchitis mainly includes inflammatory cytokine imbalance, oxidative stress, apoptosis, and prokineticin 2 (PK2)/prokineticin receptor 1 (PKR1) pathway. Increasing evidences indicate that the imbalance among these pro- and anti-inflammatory molecules, such as interleukin-1*β* (IL-1*β*), IL-6, tumor necrosis factor-*α* (TNF-*α*), and IL-10, in testicular cells can result in orchitis [3, 4]. Oxidative stress and reactive oxygen species (ROS), which are majorly sourced from macrophages [5], may be the major contributory factors leading to testicular cell apoptosis and acute inflammation of testes [6]. Additionally, it has been shown that the PK2/PKR1 pathway contributes to the development of testicular inflammation [7–9]. Hence, treatment strategies for orchitis should focus on the balance of inflammatory cytokines, removal of oxidative and apoptotic injury, and regulation of the PK2/PKR1 pathway.

Methane, the simplest alkane, is the most abundant organic compound on earth and one of the most important gases which contribute to global warming [10]. Inhaled methane limits mitochondrial electron transport chain dysfunction and confers protection against mitochondrial dysfunction during experimental liver ischemia-reperfusion injury [11]. It has been proved that inhalation of methane also modulates leukocyte activation, affects key events of ischemia-reperfusion-induced oxidative stress, and has anti-inflammatory effects against ischemia-reperfusion damage [12]. Additionally, recent researches have demonstrated that methane exerts microcirculatory and mitochondrial effects in inflammatory states [13, 14]. Methane-rich saline (MS), which is safer and more portable than methane gas, also has powerful anti-inflammatory, antioxidative, and anti-apoptotic properties [15]. Recent studies have confirmed that MS has protective effects and alleviates the inflammatory, oxidative, and apoptotic injury in many diseases, including acute pancreatitis [10], spinal cord injury [16, 17], acute lung injury [18], and immune system disorders [18]. However, the therapeutic effects of methane on acute orchitis and the underlying mechanisms have not been clarified and need to be further investigated.

In this study, we used a lipopolysaccharide- (LPS-) induced acute orchitis rat model to investigate whether methane can play a protective role against acute orchitis and to explore its potential mechanisms. Our results indicate that methane could be a new and convenient strategy for the therapy of acute orchitis.

## 2. Materials and Methods

### 2.1. Production of MS

As previously described [18], fresh physiological saline was supersaturated with pure methane (>99.9%) for 3 h under 0.4 MPa and stored under atmospheric pressure at 4°C. The concentration of methane in the MS that was freshly prepared 1 day before animal administration was 0.99 mmol/L.

### 2.2. Animals and Tissue Preparation

Adult male Sprague-Dawley (SD) rats (weight, 180-200 g) were obtained from the animal center of the Naval Medical University. All animal experiments were approved and supervised by the Animal Care and Use Committee of the Naval Medical University (permit no. SYXK-2002-042). All rats were housed in cages under controlled conditions at 22°C with *ad libitum* access to food and water and a 12-12 h day/night cycle.

A total of 96 rats were randomly divided into four groups (*n* = 24 each) as follows: (1) Control group, no treatment; (2) MS group, intraperitoneal (i.p.) administration of MS (20 mL/kg) per 12 h; (3) LPS group, the acute orchitis model was established using LPS (5 mg/kg i.p., cat. no. L2880, Sigma-Aldrich) [7]; then, rats were administrated physiological saline (20 mL/kg i.p.) per 12 h; (4) MS-LPS group, rats were induced by LPS (5 mg/kg i.p.), then administrated MS (20 mL/kg i.p.) per 12 h as previously described [18]. After LPS injection, survival experiment was monitored every 12 h (*n* = 24) and all the surviving rats were then sacrificed to collect bilateral testes and blood for further detection at 72 h.

### 2.3. Wet/Dry (*W*/*D*) Ratio

Testicular edema was assessed as the *W*/*D* ratio. In short, blood and fluid at the surface of harvested testes (*n* = 6) were immediately absorbed by a filter paper, and the testes were weighed, dried at 65°C for 48 h, and then weighed again to calculate the *W*/*D* ratio.

### 2.4. Histological Examination

Histological examination of the testes was performed via hematoxylin and eosin (HE) staining. Following anesthesia with pentobarbital sodium (40 mg/kg i.p.), rats were perfused with 0.01 M PBS for 10 min followed by 4% paraformaldehyde (PFA) for 30 min. Harvested testes were fixed in 4% PFA for 24 h, dehydrated using a gradient alcohol series, vitrified in dimethylbenzene, and embedded in paraffin. Next, tissue blocks were sectioned at 5 *μ*m thickness, stained with HE, and observed under a light microscope at ×200 magnification (Leica Microsystems GmbH) (*n* = 6‐9).

### 2.5. Enzyme Linked Immunosorbent Assay (ELISA)

After the testes were harvested, the testicular homogenate was immediately performed in 0.01 M PBS at 4°C. The homogenate was centrifuged at 3000 g for 15 min at 4°C, and the supernatant was collected for detection. Blood was collected by inserting a No. 7 needle into the tail vein. The sample was stored at 4°C overnight and centrifuged at 3000 × g for 10 min at 4°C, and the supernatant was collected for testing. ELISA kits were used to measure the levels of IL-1*β* (cat. no. PRLB00), IL-6 (cat. no. PR6000B), TNF-*α* (cat. no. PRTA00), and IL-10 (cat. no. PR1000, all R&D Systems) in the testes and serum according to the manufacturer's protocol (*n* = 6).

### 2.6. Detection of Superoxide Dismutase (SOD) and Malondialdehyde (MDA) Activity Assay

The activity assay was performed using SOD (cat. no. MM-0386R2) and MDA (cat. no. MM-0385R2, both Jiangsu Meimian Industrial Co., Ltd.) ELISA kits following the manufacturer's protocol (*n* = 6).

### 2.7. Terminal Deoxynucleotidyl Transferase dUTP Nick-End Labeling (TUNEL) Assay

Apoptosis of the injured testes was detected via TUNEL staining at 72 h after LPS administration (*n* = 3‐5). The TUNEL kit (cat. no. MK1027, Wuhan Boster Biological Technology, Ltd.) was used to detect apoptotic cells in paraffin-embedded testes sections according to the manufacturer's instructions. Cells with green-stained nuclei were considered apoptotic cells. Fluorescence intensity was calculated using ImageJ software (v. 1.52v).

### 2.8. Reverse Transcription-Quantitative PCR (RT-qPCR)

Total RNA (*n* = 6) was extracted from the testes using TRIzol (Invitrogen, Thermo Fisher Scientific, Inc.), and RT-qPCR was performed as previously described [19]. The primers used for PCR are presented in Table 1. GAPDH was used as an internal control to normalize gene expression levels, and the relative expression of target mRNA was calculated by the 2^-*ΔΔ*Cq^ method [20].

### 2.9. Western Blotting (WB)

WB for PK2 and PKR1 in the testes (*n* = 6) was performed as previously described [19]. Membranes were blocked in 5% skimmed milk/PBS for 1 h at 4°C and incubated with the following primary antibodies: rabbit polyclonal anti-PK2 (cat. no. ab76747, 1 : 200, Abcam), rabbit polyclonal anti-PKR1 (cat. no. APR-041, 1 : 200, Alomone Labs), and mouse monoclonal anti-GAPDH (cat no. WB2197, 1 : 2000, Well Biotech Co., Ltd.) at 4°C overnight and then incubated with horseradish peroxidase- (HRP-) labeled goat anti-rabbit secondary antibody (cat. no. 111-035-003, 1 : 5000, Jackson ImmunoResearch Laboratories, Inc.) at 4°C for 1 h.

### 2.10. Immunohistochemistry (IHC)

IHC was performed for PK2 and PKR 1 as previously described [19]. The primary antibodies were rabbit polyclonal anti-PK2 (cat. no. ab76747, 1 : 100, Abcam) and rabbit polyclonal anti-PKR1 (cat. no. APR-041, 1 : 100, Alomone Labs). Images were captured under a microscope at magnification ×400 (Leica Microsystems GmbH) (*n* = 3–5).

### 2.11. Statistical Analysis

All the aforementioned assays were performed at least in triplicate and were repeated at least thrice. Results were expressed as the mean ± standard deviation and were analyzed using SPSS v.21.0 software (IBM Corp.). Figures were generated using Prism v.6.0 software (GraphPad Software, Inc.). Multiple comparisons were assessed using one-way analyses of variance (ANOVA) followed by Tukey's *post hoc* tests. *P* < 0.05 was considered to indicate a statistically significant difference.

## 3. Results

### 3.1. Methane Improves the Survival Rate of LPS-Induced Acute Orchitis Rats

**Figure 1:**
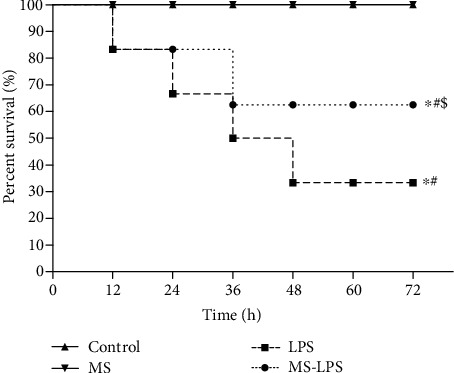
Methane improved the survival rate of LPS-induced acute orchitis rats. The survival rate was assessed every 12 h up to 72 h following exposure to LPS. ^∗^*P* < 0.05 vs the Control group, ^#^*P* < 0.05 vs the MS group, and ^$^*P* < 0.05 vs the LPS group.

Acute orchitis rats manifested lethargy, diarrhea, ruffled pelage, and conjunctival hemorrhage 3 h immediately after LPS injection. There was no significant difference in the survival rate between the Control and MS groups, meaning that methane had no significant effect on normal rats. However, the survival rate in the LPS and MS-LPS groups was significantly lower than that in the Control and MS groups (*P* < 0.05), suggesting that the survival rate was reduced by the LPS administration. Additionally, there were more surviving rats in the MS-LPS group than in the LPS group (*P* < 0.05, Figure 1). This observation indicates that methane improves the survival rate of LPS-induced acute orchitis rats.

### 3.2. Methane Decreases the *W*/*D* Ratio in the Testes of Rats with LPS-Induced Acute Orchitis

**Figure 2:**
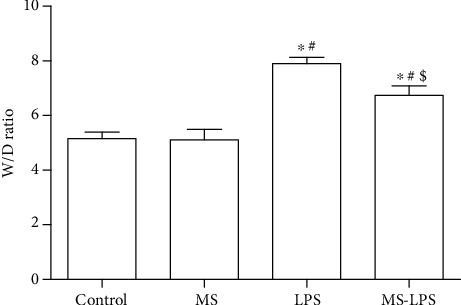
Methane decreased the *W*/*D* ratio in the testes of rats with LPS-induced acute orchitis. ^∗^*P* < 0.05 vs the Control group, ^#^*P* < 0.05 vs the MS group, and ^$^*P* < 0.05 vs the LPS group.

As shown in Figure 2, the *W*/*D* ratio in the Control group was similar to that in the MS group, whereas the *W*/*D* ratio in the LPS and MS-LPS groups was significantly higher than that in the Control and MS groups (*P* < 0.05), indicating that LPS challenge could increase the *W*/*D* ratio and damage the testes. Furthermore, the *W*/*D* ratio in the MS-LPS group was much lower than that in the LPS group (*P* < 0.05). This finding suggests that methane significantly decreases the *W*/*D* ratio of LPS-induced acute orchitis rats.

### 3.3. Methane Alleviates LPS-Induced Histological Changes in the Testes

**Figure 3:**
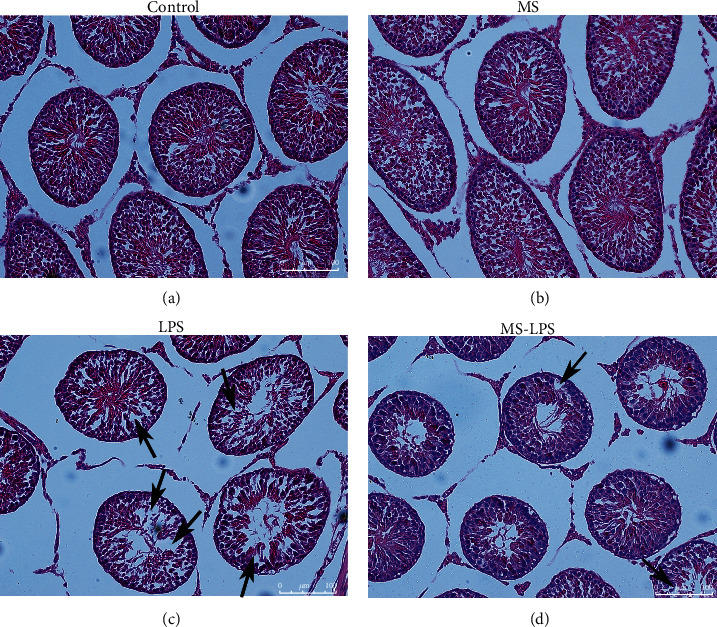
Methane alleviated LPS-induced histological changes in the testes of rats with LPS-induced acute orchitis. Spermatogenic cells were arranged in a concentric distribution in the Control (a) and MS (b) groups. Deleterious effects appeared in the LPS (c) and MS-LPS (d) groups at 72 h following LPS administration, including loss of germ cells, disorganization, and disruption of spermatogenic cells (black arrows). Scale bar = 100 *μ*m.

In the Control and MS groups, spermatogenic cells were arranged in a concentric distribution (Figures 3(a) and 3(b)). However, there appeared to be some deleterious effects in the LPS and MS-LPS groups at 72 h after LPS administration, including loss of germ cells and disorganization and disruption of spermatogenic cells (Figures 3(c) and 3(d), black arrow). In addition, there was less testicular damage in the MS-LPS group than in the LPS group, meaning that methane alleviated LPS-induced histological changes in the testes.

### 3.4. Methane Suppresses and Promotes the Production of Pro- and Anti-inflammatory Cytokines, Respectively, in the Testes and Serum following LPS Administration

**Figure 4:**
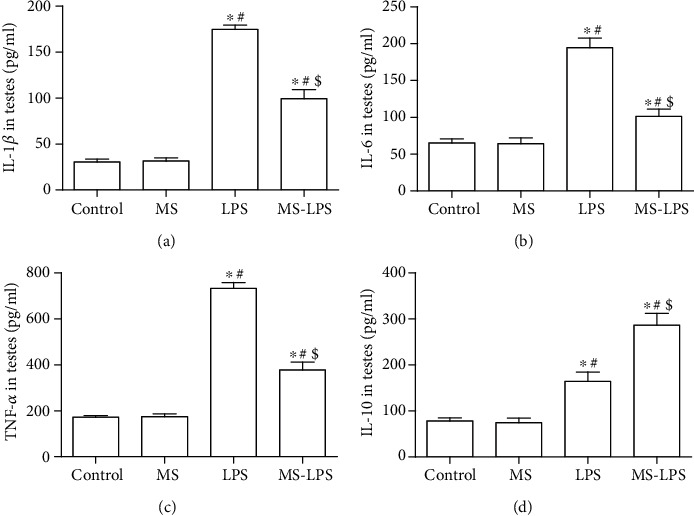
Methane suppressed the production of IL-1*β* (a), IL-6 (b), and TNF-*α* (c) and promoted the production of IL-10 (d) in the testes following LPS administration. ELISA was used to measure the production of inflammatory cytokines in the testes. ^∗^*P* < 0.05 vs the Control group, ^#^*P* < 0.05 vs the MS group, and ^$^*P* < 0.05  vs the LPS group.

**Figure 5:**
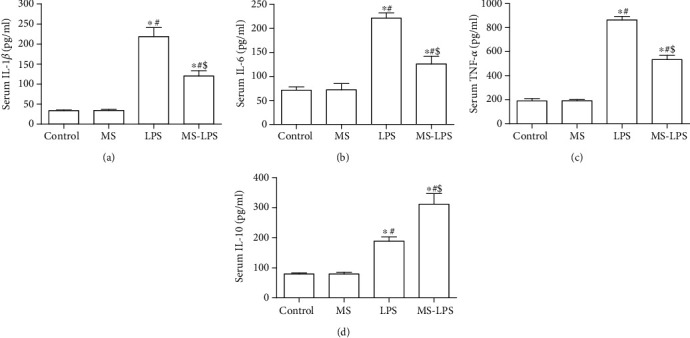
Methane suppressed the production of IL-1*β* (a), IL-6 (b), and TNF-*α* (c) and promoted the production of IL-10 (d) in serum following LPS administration. ELISA was used to measure the production of inflammatory cytokines in serum. ^∗^*P* < 0.05 vs the Control group, ^#^*P* < 0.05 vs the MS group, ^$^*P* < 0.05 vs the LPS group.

ELISA was used to detect the production of cytokines in the testes and serum following LPS administration. It was found that there was no significant difference in the production of cytokines in the testes and serum between the Control and MS groups (Figures 4 and 5). Proinflammatory cytokines, IL-1*β*, IL-6, and TNF-*α*, in the testes and serum were apparently increased in the LPS group compared with those in the Control and MS groups (*P* < 0.05), whereas their levels in the LPS group were also higher than those in the MS-LPS group (*P* < 0.05). Furthermore, the level of the anti-inflammatory cytokine, IL-10, was also higher in the LPS group than that in the Control and MS groups, while it was lower than that in the MS-LPS group (*P* < 0.05).

**Figure 6:**
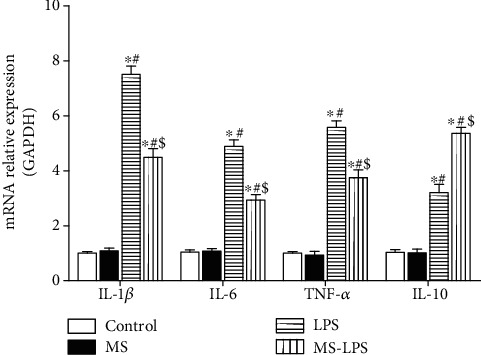
Methane suppressed the expression of IL-1*β*, IL-6, and TNF-*α* and promoted the expression of IL-10 in the testes at transcription level after LPS administration. ^∗^*P* < 0.05 vs the Control group, ^#^*P* < 0.05 vs the MS group, and ^$^*P* < 0.05 vs the LPS group.

RT-qPCR was also performed to test the expressions of IL-1*β*,IL-6, TNF-*α*, and IL-10 in the testes at transcription level in these four groups (Figure 6). The expressions of these cytokines in the Control group were similar to those in the MS group. The MS-LPS group expressed more IL-1*β*, IL-6, and TNF-*α* than the Control and MS groups, but less than the LPS group (*P* < 0.05). Additionally, the expression of IL-10 in the LPS group was higher than that in the Control and MS groups, but lower than that in the MS-LPS group (*P* < 0.05).

These above observations indicate that methane suppresses and promotes the production of pro- and anti-inflammatory cytokines, respectively, in the testes and serum after LPS administration, suggesting that methane has an anti-inflammatory effects on LPS-induced acute orchitis.

### 3.5. Methane Exerts Antioxidative Effects on the Testes after LPS Injection

**Figure 7:**
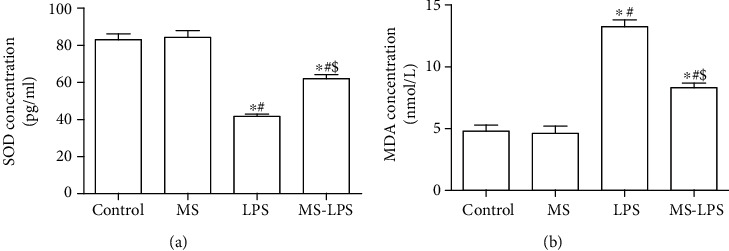
Methane increased SOD (a) and decreased MDA (b) levels in the testes of rats with LPS-induced acute orchitis. ^∗^*P* < 0.05 vs the Control group, ^#^*P* < 0.05 vs the MS group, ^$^*P* < 0.05 vs the LPS group.

Both SOD and MDA are two common representatives of oxidant-induced injury. It was found that the SOD levels in the LPS and MS-LPS groups were lower than those in the Control and MS groups, whereas the SOD level in the LPS group was much lower than that in the MS-LPS group (*P* < 0.05, Figure 7(a)). The MDA levels in the LPS and MS-LPS groups were higher than those in the Control and MS groups, while the MDA level in the LPS group was higher than that in the MS-LPS group (*P* < 0.05, Figure 7(b)). Additionally, there was no significant difference in the levels of SOD and MDA between the Control and MS groups (*P* > 0.05). These results reveal that methane increases and decreases the levels of SOD and MDA, respectively, in the testes, suggesting that methane exerts antioxidative effects on the testes after LPS injection.

### 3.6. Methane Has Antiapoptotic Effects on the Testes following LPS Administration

**Figure 8:**
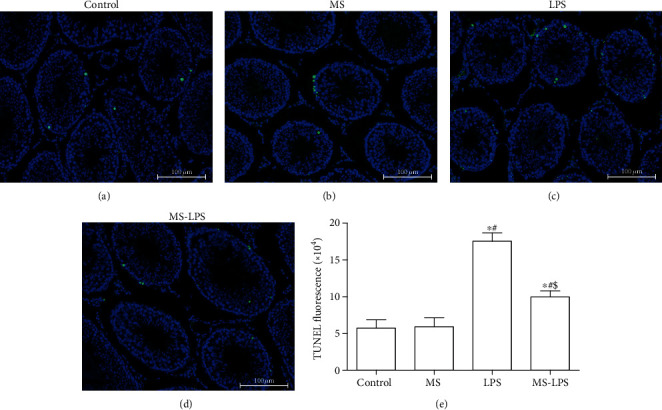
Methane had antiapoptotic effects on the testes of rats with LPS-induced acute orchitis. TUNEL assay was performed to detect the apoptosis of testes in the Control (a), MS (b), LPS (c), and MS-LPS (d) groups. Statistical analysis of TUNEL fluorescence was shown in (e). Nuclei were stained blue by DAPI, and apoptotic cells were stained with green. ^∗^*P* < 0.05 vs the Control group, ^#^*P* < 0.05 vs the MS group, and ^$^*P* < 0.05 vs the LPS group. Scale bar = 100 *μ*m.

The TUNEL assay was performed to detect apoptosis in the testes following LPS administration (Figure 8). There were a few TUNEL-positive cells in the Control and MS groups, meaning that only a few apoptotic cells in the testes from the Control and MS groups (Figures 8(a) and 8(b)). The number of TUNEL-positive cells in the LPS and MS-LPS groups was larger than that in the Control and MS groups (*P* < 0.05), suggesting that there were more apoptotic cells in the testes following LPS injection. Furthermore, there were more apoptotic cells in the LPS group than in the MS-LPS group (*P* < 0.05). This result indicates that methane has antiapoptotic effects on the testes following LPS administration.

### 3.7. Methane Decreases PK2 and PKR1 Expressions in the Testes of LPS-Induced Acute Orchitis Rats

**Figure 9:**
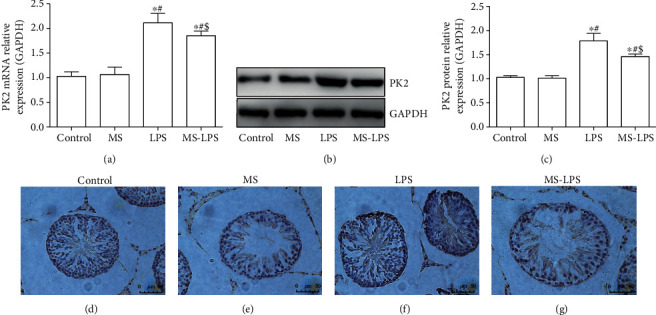
Methane decreased PK2 expression in the testes of rats with LPS-induced acute orchitis. RT-qPCR (a), WB (b, c), and IHC (d–g) were used to determine the expression of PK2 in the testes at 72 h after LPS injection. ^∗^*P* < 0.05 vs the Control group, ^#^*P* < 0.05 vs the MS group, ^$^*P* < 0.05 vs the LPS group. Scale bar = 50 *μ*m.

To investigate the underlying mechanisms, RT-qPCR, WB, and IHC were used to determine the expression of PK2 and PKR1 in the testes at 72 h after LPS administration. It was shown that the expression of PK2 at the transcription level in the Control group was in line with that in the MS group, whereas it was increased in the LPS and MS-LPS groups (*P* < 0.05, Figure 9(a)). The expression of PK2 at the protein level which was obtained by WB (Figures 9(b) and 9(c)) and IHC (Figures 8(d–g)) was similar to the results of RT-qPCR. There was no significant difference in the expression of PK2 protein between the Control and MS groups (*P* > 0.05). The expression of PK2 protein in the LPS and MS-LPS groups was approximately 1.78 and 1.46 times of that in the Control group, respectively (*P* < 0.05). Additionally, it was also found that the LPS group expressed more PK2 at transcription and protein levels than the MS-LPS group (*P* < 0.05).

**Figure 10:**
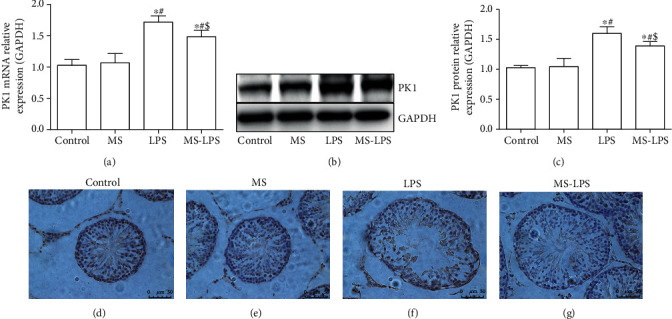
Methane decreased the expression of PKR1 in testes of rats with LPS-induced acute orchitis. RT-qPCR (a), WB (b-c), and IHC (d-g) were used to determine the expression of PKR1 in the testes at 72 h after LPS injection. ^∗^*P* < 0.05 vs the Control group, ^#^*P* < 0.05 vs the MS group, ^$^*P* < 0.05 vs the LPS group. Scale bar = 50 *μ*m.

The expression of PKR1 in the testes was also determined 72 h after LPS administration. It was revealed that the expression of PKR1 in the LPS and MS-LPS groups was much higher than that in the Control and MS groups at transcription and protein levels (*P* < 0.05, Figure 10). The expression of PKR1 in the MS-LPS group was much lower than that in the LPS group (*P* < 0.05). Additionally, there was no apparent difference in the expression of PKR1 between the Control and MS groups (*P* > 0.05).

These above observations suggest that methane decreases PK2 and PKR1 expressions in the testes of rats with LPS-induced acute orchitis.

## 4. Discussion

In the present study, it was found that methane improved the survival rate and had anti-inflammatory, antioxidative, and antiapoptotic effects on the testes of LPS-induced acute orchitis rat models. Additionally, it was also proved that methane decreased the PK2 and PKR1 expressions in the testes. These observations revealed that methane had therapeutic effects on acute orchitis via regulation of the PK2/PKR1 pathway.

As the simplest alkane, methane has been used as gas fuel by human for hundreds of years and plays an important role in global warming [10]. Methane and hydrogen gas are products of bacterial metabolism in human gut, whereas hydrogen can also be converted to methane by methane-producing bacteria [21, 22]. In recent decades, researchers have focused on the physiological and therapeutic properties of methane. It has been confirmed that methane exerts anti-inflammatory, antioxidative, and antiapoptotic effects and influences several pathological processes in various diseases [10, 15–17, 23–25]. What is more, as a gas molecule, methane can easily penetrate cell membranes and organelles and persist in tissues owing to its lipid-soluble peculiarity. These features of methane provide the possibility of being used for the therapy of acute orchitis.

Inflammation, characterized by inflammatory cytokine imbalance, plays a vital role in the pathogenesis of acute orchitis. It has been proved that testicular cells, including the Sertoli cells and Leydig cells, can secrete inflammatory cytokines in response to LPS [3]. Additionally, increased circulating leukocytes within the testis might contribute to the production of inflammatory mediators. Here, we found that the production of pro- and anti-inflammatory cytokines at transcription and protein levels increased in not only serum but also the testes of LPS-challenged rats. It was also revealed that the MS-LPS group produced less IL-1*β*, IL-6, and TNF-*α* and more IL-10 than the LPS group. These observations are consistent with other reports [15, 26] and suggest that methane suppresses and promotes the production of pro- and anti-inflammatory cytokines, respectively, and exerts protective roles in LPS-induced acute orchitis rats.

Oxidative stress, especially ROS, which can induce tissue and cell damage is central to orchitis pathology and is known to mediate testicular damage [27]. Excessive ROS production directly induces lipid peroxidation and mitochondrial lesions in germ cells and leads to dysfunctional spermatogenesis [28]. Additionally, increased ROS production exerts genotoxic effects that lead to DNA damage, germ cell abnormalities, and an unbearable state of antioxidative defense [29, 30]. Therefore, increased ROS generation and reduction of antioxidative defense system play significant roles in LPS-induced orchitis. SOD is a critical enzyme in the antioxidative defense system and eliminates ROS production which generated from H_2_O_2_ and oxygen [6]. The lipid peroxidation biomarker, MDA, is produced from cell and mitochondrial membrane lipid peroxidation and directly reflects the amount and activity of ROS *in vivo* [31]. In the present study, it was revealed that the SOD level decreased and the MDA level increased in the LPS and MS-LPS groups after LPS administration, whereas the SOD level was higher and the MDA level was lower in the MS-LPS group than in the LPS group. These data suggest that methane has antioxidative effects on LPS-induced acute orchitis which is similar to other studies [15, 23, 32].

Apoptosis which occurs in multicellular organisms is a process of programmed cell death and is important for homeostasis in multicellular life forms [32]. Inflammation and increased ROS production can lead to testicular cell apoptosis in an acute orchitis rat model. There were more TUNEL-positive cells in the LPS and MS-LPS groups than in the Control and MS groups, indicating that apoptosis was increased at 72 h following LPS administration. Additionally, there were fewer apoptotic cells in the MS-LPS group than in the LPS group. This reveals that methane has antiapoptotic effects on acute orchitis.

In recent years, several studies have focused on the molecular mechanisms of methane. It has been proved that methane inhibits NF-*κ*B MAPK's pathway, enhances PI3K-Akt-GSK3*β*-mediated IL-10 expression, and then exerts anti-inflammatory effects [33, 34]. Others have demonstrated that methane has anti-inflammatory, antioxidative, and antiapoptotic effects via Nrf2 activation in spinal cord ischemia-reperfusion injury [17] and via regulation of endoplasmic reticulum stress in sepsis-induced acute kidney injury [32]. However, the exact underlying mechanisms of the protective roles of methane have not been fully clarified.

PK2 exerts chemokine function by binding with cognate G-protein-linked receptors [8] and is involved in multiple physiological and pathological processes, including reproduction, angiogenesis, and neurogenesis [35–38]. PK2 also participates in inflammation and plays an important role in inducing macrophage infiltration and proinflammatory cytokine release [39]. Moreover, PK2 is highly expressed in mammalian testes [40] and the PK2/PKR1 pathway contributes to the development of LPS-induced orchitis [7] and experimental autoimmune orchitis [8]. Here, the expressions of PK2 and PKR1, as well as IL-1*β*, IL-6, and TNF-*α*, increased after LPS injection, which is consistent with the fact that PK2 enhances the LPS-induced production of proinflammatory cytokines [39]. In addition, the expressions of PK2 and PKR1 in the MS-LPS group are lower than those in the LPS group. We speculate that methane exerts anti-inflammatory, antioxidative, and antiapoptotic effects against LPS-induced acute orchitis which may be via regulation of the PK2/PKR1 pathway.

In the present study, we have demonstrated that methane is beneficial for anti-inflammation, antioxidant, and antiapoptosis in LPS-induced acute orchitis. Furthermore, we have also revealed the underlying mechanism of the therapeutic effects of methane in acute orchitis through the regulation of the PK2/PKR1 pathway. However, the limitations of this study include a single dose of MS administration and lack of knowledge about the detailed molecular mechanisms. Further investigations will be performed to clarify whether methane has therapeutic effects in a dose-dependent manner and to fully understand its mechanism in detail.

## 5. Conclusion

Methane has anti-inflammatory, antioxidative, and antiapoptotic effects against LPS-induced acute orchitis via regulation of the PK2/PKR1 pathway. Therefore, methane could be a new and convenient strategy for the treatment of inflammation-related testicular diseases.

## Figures and Tables

**Table 1:** Sequences of primers utilized for RT-qPCR analysis.

Target	Forward(5′-3′)	Reverse(5′-3′)
GAPDH	GAAGGGCTCATGACCACAGT	GGATGCAGGGATGATGTTCT
IL-1*β*	CTCACAGCAGCATCTCGACAAGAG	TCCACGGGCAAGACATAGGTAGC
IL-6	ACTTCCAGCCAGTTGCCTTCTTG	TGGTCTGTTGTGGGTGGTATCCTC
TNF-*α*	ATGGGCTCCCTCTCATCAGTTCC	GCTCCTCCGCTTGGTGGTTTG
IL-10	CTGCTCTTACTGGCTGGAGTGAAG	TGGGTCTGGCTGACTGGGAAG
PK2	GCTACCGCTGCTGCTCACAC	CCTCCTCCACACTGAGAGTCCTTG
PKR1	TGTGCTGGGCTCCCTTCTATGG	AGGCGGTGAGGTAGTGCTTCTC

## Data Availability

The data used to support the findings of this study are available from the corresponding author upon request.
